# Integrative transcriptomic meta-analysis of Parkinson’s disease and depression identifies *NAMPT* as a potential blood biomarker for de novo Parkinson’s disease

**DOI:** 10.1038/srep34579

**Published:** 2016-09-29

**Authors:** Jose A. Santiago, Alyssa M. Littlefield, Judith A. Potashkin

**Affiliations:** 1 The Cellular and Molecular Pharmacology Department, The Chicago Medical School, Rosalind Franklin University of Medicine and Science, North Chicago, IL, USA

## Abstract

Emerging research indicates that depression could be one of the earliest prodromal symptoms or risk factors associated with the pathogenesis of Parkinson’s disease (PD), the second most common neurodegenerative disorder worldwide, but the mechanisms underlying the association between both diseases remains unknown. Understanding the molecular networks linking these diseases could facilitate the discovery of novel diagnostic and therapeutics. Transcriptomic meta-analysis and network analysis of blood microarrays from untreated patients with PD and depression identified genes enriched in pathways related to the immune system, metabolism of lipids, glucose, fatty acids, nicotinamide, lysosome, insulin signaling and type 1 diabetes. Nicotinamide phosphoribosyltransferase (*NAMPT*), an adipokine that plays a role in lipid and glucose metabolism, was identified as the most significant dysregulated gene. Relative abundance of *NAMPT* was upregulated in blood of 99 early stage and drug-naïve PD patients compared to 101 healthy controls (HC) nested in the cross-sectional Parkinson’s Progression Markers Initiative (PPMI). Thus, here we demonstrate that shared molecular networks between PD and depression provide an additional source of biologically relevant biomarkers. Evaluation of *NAMPT* in a larger prospective longitudinal study including samples from other neurodegenerative diseases, and patients at risk of PD is warranted.

Parkinson’s disease (PD) is a devastating neurodegenerative disease that affects movement and it is characterized by the progressive and selective loss of nigrostriatal dopamine neurons and the presence of proteinaceous cytoplasmic inclusions called Lewy Bodies^1^. Although PD is predominantly characterized as a movement disorder, emerging research indicates that a wide range of non-motor conditions including constipation, sleep disturbances, diabetes, cognitive decline, and depression may play a role in the development of PD. Among these conditions, major depressive disorder (MDD) is one of the most common non-motor symptoms with up to 35% or more PD patients suffering from depression early in the disease^2^,^3^. Characteristic symptoms of depression including loss of appetite, sleep disturbances, fatigue, and loss of energy, are commonly observed in PD patients^4^.

Increasing evidence from epidemiological studies suggest that patients with MDD have an increased risk of PD compared to patients with other chronic conditions including osteoarthritis and diabetes^5^,^6^,^7^,^8^,^9^,^10^,^11^. More recently, a direct association between depression and subsequent development of PD was confirmed in the largest to date case control study including over 140,000 individuals with depression. Strikingly, the association between depression and PD was significant for a follow-up period of more than 2 decades suggesting that depression may be one of the earliest prodromal symptoms of PD^12^. Despite this progress, the mechanisms underlying the association between PD and depression remains poorly understood.

Diagnosis of PD and MDD relies upon the assessment of clinical symptoms and to date, there are no fully validated biomarkers for either PD or MDD. In this context, blood biomarkers are promising and several molecular signatures have been identified in blood of PD patients^13^,^14^,^15^,^16^,^17^,^18^ and MDD patients^19^,^20^,^21^ with the potential to become useful clinical diagnostics. Recently, network-based approaches have been employed to identify novel diagnostics, biological pathways and therapeutic targets for several neurodegenerative disorders^22^,^23^,^24^ and depression^19^. More relevant, network-based approaches have been used to dissect the molecular networks in PD and diabetes and to identify biologically relevant biomarkers for PD^25^,^26^,^27^,^28^. Using similar methods, herein we interrogate the blood transcriptome of untreated PD and MDD patients to identify shared dysregulated pathways and biologically relevant biomarkers. We identified nicotinamide phosphoribosyltransferase (*NAMPT*), an adipokine involved in lipid, glucose metabolism, inflammation and insulin resistance^29^, as a potential blood biomarker for de novo PD patients.

## Results

### Transcriptomic and network analyses of PD and MDD blood microarrays

**Figure 1:**
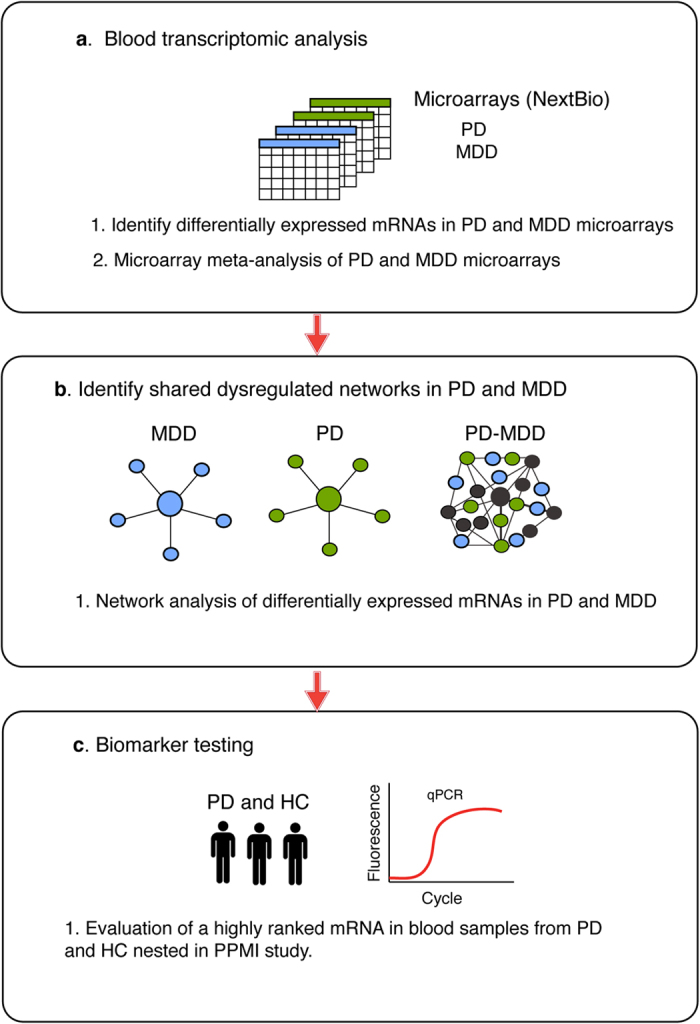
Integrative transcriptomic and network analysis. Microarray datasets from PD and MDD were downloaded from NextBio Research. Differential gene expression and microarray meta-analysis were performed using NextBio. Differentially expressed genes shared between PD and MDD were analyzed using network and pathway analysis in GeneMANIA. The most significant gene ranked in meta-analysis and in network analysis was tested in RT-qPCR assays in RNA samples from blood of early stage and drug naïve PD patients and HC nested in PPMI.

**Figure 2:**
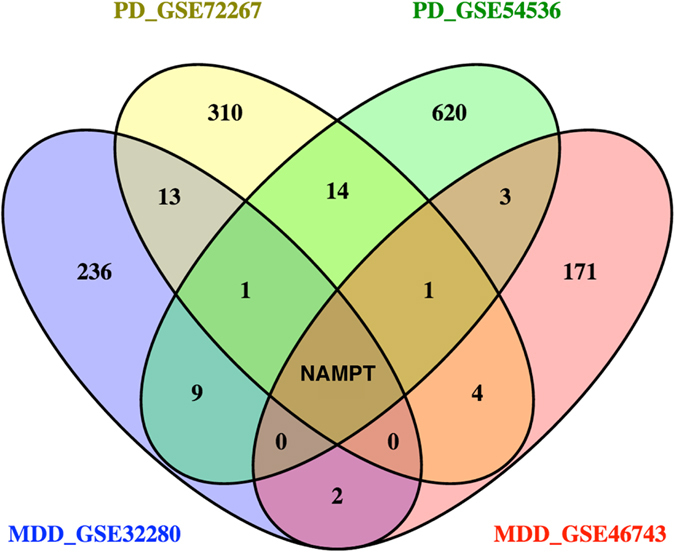
Venn diagram analysis. Venn diagram analysis of differentially expressed genes in blood microarrays of untreated PD and MDD patients identified *NAMPT* as the only overlapping gene across all four datasets.

To identify a common transcriptional signature in blood of PD and MDD patients, we searched the curated database NextBio Research for human microarray studies from untreated PD and MDD patients (See Materials and Methods). Four microarray studies met the inclusion criteria and were used for further analysis (Table 1). The overall analysis strategy is presented in Fig. 1. We first analyzed the overlap in gene expression between each individual PD and MDD datasets. Only genes with a fold change of 1.2 or higher and a p-value of less than 0.05 were included in the analysis. Using this cut-off criteria, few genes in blood were identified as overlapping between each PD and MDD dataset (Fig. 2). Venn diagram analysis identified *NAMPT* as the only overlapping gene in all four datasets of untreated PD and MDD patients (Fig. 2).

**Figure 3:**
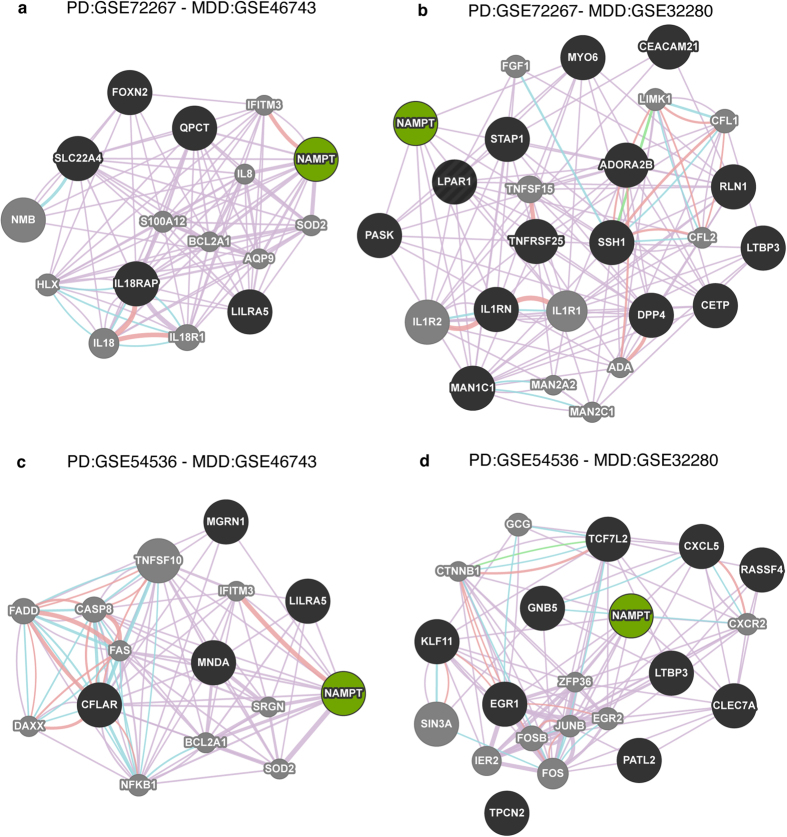
Network analysis of shared genes in PD and MDD. Gene network analysis was performed in GeneMANIA. Shared genes between PD and MDD are displayed in black circles and other genes with the greater number of interactions are displayed in gray circles. The sizes of the gray nodes represent the degree of association with the input genes (i.e, smaller size represents low connectivity). Purple lines indicate coexpression, blue lines indicate pathway, pink lines show physical interactions, green lines are genetic interactions. *NAMPT*, highlighted in green, was the only common gene across all the datasets.

In order to identify the biological and functional properties of genes shared between PD and MDD, we performed network analysis on each pair of shared genes between PD and MDD datasets using GeneMANIA^30^ (Fig. 3). Genes within these networks were enriched in pathways related to the immune system (p = 8.6E-09), adipocytokine signaling (7.4E-08), epidermal growth factor receptor pathway (EGFR) (p = 7.9E-04), and type 1 diabetes (p = 1.5E-03).

**Figure 4:**
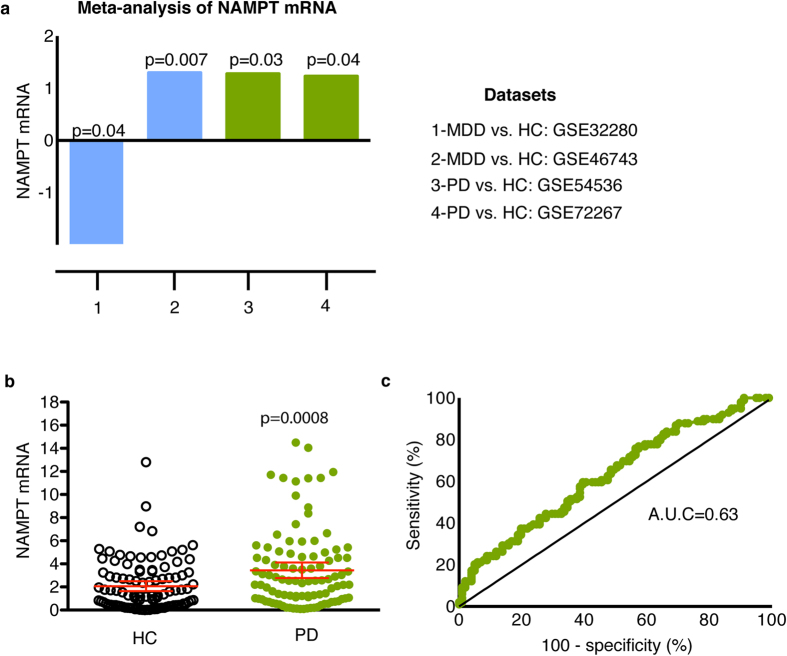
Evaluation of *NAMPT* mRNA in PD. (**a**) Meta-analysis results for *NAMPT* mRNA across the four PD (green) and MDD (blue) datasets used in this study. *NAMPT* was significantly upregulated in both datasets from untreated PD patients compared to HC. (**b**) RT-qPCR assays were used to confirm the results from the meta-analysis. Relative abundance of *NAMPT* mRNA in blood of 99 PD patients (green) compared to 101 HC (white) in samples obtained from PPMI. The geometric mean of two reference genes, *GAPDH* and *PGK1*, were used to normalize for input RNA. A Student t-test (two-tailed) was used to assess the significance between PD and controls. Error bars represent 95% confidence interval. A p-value of 0.05 or less was regarded as significant (**c**) ROC analysis of *NAMPT* resulted in an AUC value of 0.63.

We next performed an integrative meta-analysis of all datasets using the meta-analysis tool in NextBio (See Methods). *NAMPT* was identified as the most significant gene with differential expression in blood of untreated PD and MDD patients across all four datasets (Fig. 4a). Specifically, *NAMPT* was upregulated in both datasets from untreated PD patients and in one out of the two MDD datasets (Fig. 4a). The top 20 genes identified in the meta-analysis are listed in Table 2 and the complete list is provided in Supplementary Table S1.

**Figure 5:**
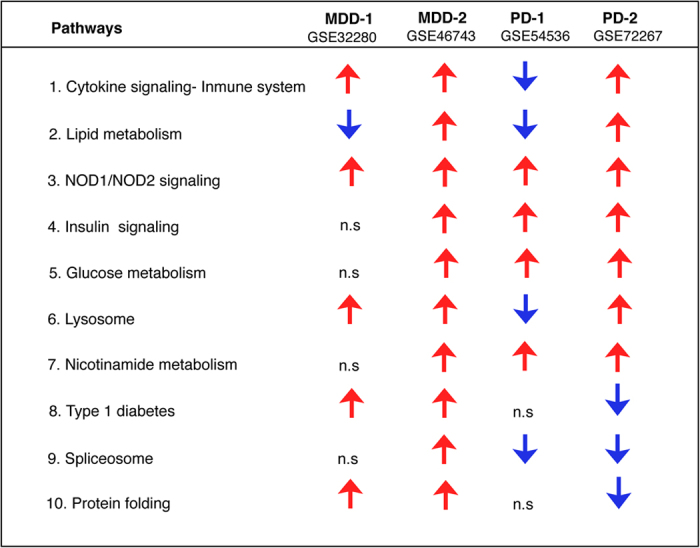
Shared dysregulated pathways in MDD and PD. Biological and functional analysis of differentially expressed genes in the MDD and PD datasets used in this study was performed using the Molecular Signatures Database (MSigDB) in NextBio. Differentially expressed genes in each dataset were enriched in the canonical pathways shown in this table (MSigDB). Red and blue arrows indicate upregulation and downregulation, respectively. n.s indicates not significant.

Biological pathways altered across the PD and MDD datasets were determined using a rank-based enrichment statistical analysis and the Molecular Signatures Database (MSigDB) in NextBio. Shared genes identified in meta-analysis were enriched in several pathways including cytokine signaling, lipid metabolism, nucleotide-binding oligomerization domain-like receptors 1 and 2 (NOD1/2) signaling, insulin signaling, glucose metabolism, lysosome, nicotinamide metabolism, type 1 diabetes, spliceosome and protein folding (Fig. 5). Most of the overrepresented biological pathways were upregulated in both MDD and PD. Genes related to splicing and protein folding appeared to be upregulated in MDD but downregulated in PD.

### Biomarker evaluation in de novo PD patients

In order to confirm the results from the meta-analysis and network analysis, we tested *NAMPT* mRNA using real time quantitative polymerase chain reaction (RT-qPCR) assays in blood samples from early stage and drug naïve PD patients and healthy controls (HC) nested in Parkinson’s Progression Markers Initiative (PPMI). Demographic and clinical characteristics of study participants are provided in Table 3. Statistical comparisons of demographic and clinical characteristics for this subset of participants have been published elsewhere^15^. Briefly, there were no significant differences in mean age and sex distribution between PD and HC (Table 3). PD patients had a small but significantly less years of education compared to HC (p = 0.02)^15^. RT-qPCR assays revealed that the relative abundance of *NAMPT* mRNA was upregulated in PD patients compared to HC (p = 0.0008) (Fig. 4b). This result was sustained after adjusting for covariates including age, sex, education and RNA integrity using a general linear model (p = 0.0006). Pearson correlation analysis demonstrated that relative abundance of *NAMPT* mRNA did not correlate with any of the clinical variables including Hoehn & Yahr (p = 0.26), Movement Disorder Society-sponsored revision of the Unified Parkinson’s Disease Rating Scale (MDS-UPDRS) total (p = 0.86), MDS-UPDRS part I (p = 0.24), MDS-UPDRS part I patient questionnaire (p = 0.21), MDS-UPDRS part II patient questionnaire (p = 0.80), MDS-UPDRS part III patient questionnaire (p = 0.93), and University of Pennsylvania Smell Identification Test (UPSIT) (p = 0.24). Correlation of *NAMPT* mRNA with the Geriatric depression scale (GDS) trended toward significance but was weak (r = 0.13, p = 0.07). Receiver operating characteristic curve (ROC) analysis resulted in an area under the curve (AUC) value of 0.63 (Fig. 4c).

### Building a non-invasive diagnostic model for PD

We next sought to build a non-invasive diagnostic model for PD by integrating the results from our RNA biomarkers with the UPSIT clinical test, which has been shown to be a highly predictive indicator of neurodegeneration^31^. UPSIT is a commercially available test that consists of a scratch and sniff exam, which can be self-administered to test an individual’s olfactory function^32^. We performed a forward step-wise linear discriminant analysis to achieve the highest sensitivity and specificity (Methods). We first combined our RNA biomarkers including *NAMPT* and the coatomer protein complex subunit zeta 1 (*COPZ1*), a blood RNA biomarker replicated previously in the same subset of samples from Parkinson’s Progression Markers Initiative (PPMI)^15^. Using both markers individually and in combination resulted in an overall diagnostic accuracy of 58% and *COPZ1* was removed from the model (Supplementary Tables S2–S5).

We next combined both RNA markers with UPSIT scores. Based on this analysis, UPSIT and *NAMPT* mRNA were capable to distinguish PD patients from HC, independently of sex and age, with an overall diagnostic accuracy of 86% (90% sensitivity, 82% specificity) (Supplementary Tables S6 and S7). *COPZ1*, age, and sex were excluded from the model. Using UPSIT scores alone, PD patients were identified with an overall diagnostic accuracy of 84% (91% sensitivity, 80% specificity) (Supplementary Table S8), indicating the limited contribution of *NAMPT* mRNA to the classification model.

## Discussion

Mounting evidence suggests that depression plays an important role in the pathogenesis of PD. Despite the increasing evidence from epidemiological studies, the molecular mechanisms linking both diseases remain unknown. Several hypotheses have been proposed to explain the relationship between depression and PD. For example, the “serotonin hypothesis” is based upon the finding that serotonin activity is lower in the brains of patients with depression and PD compared to healthy individuals^33^,^34^. Another hypothesis, the Braak hypothesis, states that alpha synuclein, a central protein in the pathogenesis of PD, is sequentially accumulated in the raphe nuclei, where serotonin is released, and later in the substantia nigra, where dopamine neurons control movement^35^. Lastly, it has been proposed that proinflammatory cytokines cause alterations in serotonin and dopamine neurotransmission leading to depression and PD^36^. Despite the accumulating evidence, the precise mechanism underlying the association between depression and PD remains unknown. Therefore, a system-level understanding of PD and depression may lead to novel diagnostic and therapeutic approaches.

To this end, we employed an integrated transcriptomic and network analysis to identify shared dysregulated pathways and molecular networks in MDD and PD. Because drugs to treat PD or depression may affect gene expression changes in blood, we used microarray datasets from drug naïve PD and MDD patients. Network analysis revealed that shared genes between PD and MDD datasets were enriched in pathways related to the immune system, adipocytokine signaling, EGFR pathway, and type 1 diabetes. In this context, growing evidence suggests that inflammation and diabetes may be involved in the pathogenesis of PD^27^,^28^,^37^,^38^,^39^. Similarly, increased inflammation has been associated with decreased corticostriatal functional connectivity in depression^40^ and elevated levels of inflammatory cytokines including interleukins IL-6, IL-1β, and tumor necrosis factor (TNF) have been found in serum of MDD patients compared to non-depressed subjects^41^. Low plasma levels of epidermal growth factor (EGF) have been associated with cognitive decline in PD patients^42^,^43^. Likewise, increased plasma levels of EGF have been found in MDD patients compared to non-depressed controls^21^. Thus, EGF may be a useful biomarker for PD and depression.

We next performed an integrative transcriptomic meta-analysis of four blood microarrays from untreated PD and MDD patients. Consistent with the results from the network analysis, genes identified in the meta-analysis were enriched in several pathways including cytokine signaling, lipid metabolism, NOD1/2 signaling, insulin signaling, glucose metabolism, lysosome, nicotinamide metabolism, type 1 diabetes, spliceosome and protein folding (Fig. 5). Most of these pathways appeared to be dysregulated in the same direction in both PD and MDD, thus reinforcing the numerous epidemiological studies that have shown a positive association between both diseases (Fig. 5)^5^,^6^,^7^,^8^,^9^,^10^,^11^. Notably, NOD1/2 signaling, important for the induction of inflammatory processes, is upregulated across all datasets. This is not surprising since the increased expression levels of inflammatory molecules are prominent features of both PD and MDD and are thought to play a causative role in both diseases^39^,^40^,^41^.

Genes involved in protein misfolding, a central mechanism in the pathogenesis of PD, appeared to be upregulated in MDD datasets but downregulated in PD. To the best of our knowledge, dysregulation of this pathway has not been documented in MDD. Similarly, genes involved in the spliceosome were upregulated in MDD and downregulated in PD (Fig. 5). In this context, aberrant splicing has been implicated in both PD^13^,^14^,^22^,^44^,^45^ and MDD^46^. Nonetheless, the pathway divergence observed in protein folding and splicing in MDD and PD warrants further investigation.

Meta-analysis identified *NAMPT* mRNA as the most significant gene dysregulated in blood of PD and MDD patients. Specifically, *NAMPT* mRNA was significantly upregulated in blood of PD patients compared to HC in both datasets from PD patients. NAMPT is a regulator of the intracellular nicotinamide adenine dinucleotide (NAD), an essential coenzyme involved in the cellular oxidative stress response. Recently, treatment with an enzymatic product of NAMPT protected against 6-hydroxydopamine (6-OHDA) neurotoxicity *in vitro*, thus suggesting a novel therapeutic strategy for PD^47^. Interestingly, altered levels of extracellular NAMPT are associated with several metabolic conditions including obesity, non-alcoholic fatty liver disease, and type 2 diabetes^29^. Further, NAMPT is an adipocytokine secreted by visceral fat tissues with insulin-mimetic effects^48^ and its mRNA expression is stimulated by factors associated with insulin resistance such as IL-6, dexamethasone, growth hormone, and TNF^29^. In this regard, insulin resistance has been associated with PD^27^,^28^ and drug naïve PD patients have been found to have glucose levels characteristic of insulin resistance^15^. Recently, several studies have identified genetic overlap between diabetes and MDD^49^,^50^. Thus, diabetes may play an important role in the pathogenesis of both PD and MDD.

We next evaluated *NAMPT* as a potential biomarker for PD using blood samples from PD patients and HC nested in PPMI. Relative abundance of *NAMPT* levels were significantly increased in early stage drug naïve PD patients compared to HC, although a substantial overlap in expression levels between the two groups was observed. The AUC value assessed by ROC curve analysis was 0.63 thus demonstrating a low diagnostic capacity. Nonetheless, this diagnostic capacity is similar to other RNA biomarkers that have been tested in blood of untreated PD patients. For instance, relative abundance of *COPZ1* and synuclein alpha (SNCA) mRNAs were differentially expressed in PD patients compared to HC nested in PPMI^15^,^51^. The reported AUC values for *COPZ1* and *SNCA* in PPMI were 0.60 and 0.58, respectively. Besides RNA markers, reduced plasma levels of apolipoprotein A1 (APOA1) were confirmed in PPMI^17^. Despite this progress, none of these biomarkers have achieved the optimal diagnostic capacity to be translated into the clinical setting.

Integration of omics approaches with clinical information has the potential to improve the diagnosis of PD. Recently, an integrative model including genetic risk factors, demographic information and olfactory function using the UPSIT scores, correctly distinguished early stage untreated PD patients from HC nested in PPMI with 83% sensitivity and 90% specificity^31^. Of note, the classification model using UPSIT scores alone was highly accurate compared to the integrative model^31^. Similarly, we combined our biomarker expression data with UPSIT scores and achieved comparable results. Our classification model including UPSIT scores and *NAMPT* mRNA were capable to distinguish PD patients from HC with 90% sensitivity and 82% specificity. Nonetheless, using UPSIT scores alone PD patients were classified with 91% sensitivity and 80% specificity thereby demonstrating the limited contribution of *NAMPT* mRNA to the model. In this study, like the integrative model proposed by Nalls *et al*.^31^, UPSIT test alone is individually strong to distinguish PD patients from HC. Despite the high diagnostic accuracy afforded by UPSIT, olfactory dysfunction is present in atypical parkinsonian disorders and other neurodegenerative diseases including Alzheimer’s disease and cerebellar ataxia^52^,^53^. Thus, olfactory dysfunction is not restrictive to PD and therefore, UPSIT analysis alone is not specific enough to overcome the high misdiagnosis rate in PD and other neurodegenerative disorders.

The search for a non-invasive biomarker with the optimal sensitivity and specificity continue to be a major challenge in the field. We expect that a combination of protein and RNA markers will significantly improve the diagnosis of untreated PD patients. In addition, it will be important to evaluate *NAMPT* mRNA in larger prospective longitudinal studies and in at risk populations for PD. Further, *NAMPT* may be an early indicator of neurodegeneration in MDD patients. Therefore, future studies will seek to evaluate *NAMPT* in blood of MDD patients and PD patients with comorbid MDD.

## Methods

### Microarray meta-analysis and network analysis

We used the curated database NextBio Research (Illumina Inc, CA, USA) to search gene expression studies in PD and MDD. Microarray studies using RNA prepared from human blood from untreated PD, MDD patients and healthy controls at baseline were used for subsequent analysis. Using the search terms “Parkinson’s disease”, “blood”, “depression”, “major depression disorder”, “transcriptional profiling” we identified 4 microarrays studies that meet our inclusion criteria as of March 01, 2016. Description of microarray datasets included in this study is provided in Table 1. Differentially expressed genes were extracted from NextBio. Negative values, if any, were replaced with the smallest positive number in the dataset. Statistical analyses were performed on log scale data. Genes whose mean normalized test and control intensities were both less than the 20th percentile of the combined normalized signal intensities were removed. Microarray meta-analysis was performed for PD and MDD datasets using the meta-analysis tool in NextBio that uses a normalized ranking approach, which enables comparability across gene expression datasets from different studies, platforms, and methods by removing dependence on absolute values of fold changes^54^,^55^. Ranks are assigned to each gene signature based on the magnitude of fold-change and then normalized to eliminate any bias owing to varying platform size. Only genes with a p-value of 0.05 or less and an absolute fold-change of 1.2 or greater were regarded as significantly differentially expressed. This meta-analysis tool has been used by others to identify dysregulated pathways shared in mouse and human studies^55^. Network analysis was performed using GeneMANIA^30^ in Cytoscape v.3.0.3. We used the default settings to include the 10 genes that have the highest number of interactions and advanced settings to include co-expression, physical, genetic, pathways, and transcription factor interactions.

### Study Participants

The parent cohort for this study is the PPMI, a 5-year observational, longitudinal, and international study comprising over 400 untreated and early stage PD patients and nearly 200 HC recruited from 32 clinical sites in Australia, United States of America (USA) and Europe. A power analysis based on each biomarker from our previous studies^15^,^22^,^25^, indicated that a minimum fold change of 1 between PD and controls could be detected with a 95% power using 100 samples per group. We selected a total of 200 participants including 99 PD patients and 101 age and sex matched HC nested in the PPMI study. PD patients that had a dopamine transporter deficit assessed by DaTscan imaging and with a Hoehn and Yahr stage I or II were chosen for this study. HC were cognitively normal, free of neurological disorder, and with no detectable dopamine transporter deficit. Demographic and clinical characteristics about the study participants are shown in Table 3. This table has been slightly modified from our previous publication^15^. Methods were conducted in accordance with the rules and guidelines of The Institutional Review Boards of Rosalind Franklin University of Medicine and Science. The Institutional Review Boards of Rosalind Franklin University of Medicine and Science and all sites participating in the PPMI study approved the study. The list of all participant clinical sites can be found at the PPMI website (http://www.ppmi-info.org/about-ppmi/ppmi-clinical-sites/). Written informed consent was obtained from all participants before inclusion in the study. All participants were evaluated for clinical features by investigators at each participant site. More information about study participants have been described elsewhere in refs. 15 and 56 and at the PPMI website (http://www.ppmi-info.org/). To access the data from this manuscript or to request samples visit the PPMI website (http://www.ppmi-info.org/access-data-specimens/request-specimens/).

### Blood sample collection and handling

Whole blood was collected at baseline during 8 am–10 am, at each participant site as described in the PPMI biologics manual (http://www.ppmi-info.org/). Patients were asked to fast overnight before blood collection. PAXgene blood RNA tubes were used according to the study protocol as described in the PPMI biologics manual and elsewhere in ref. 15. Briefly, PAXgene tubes containing blood were immediately inverted gently 8–10 times to mix the samples. Tubes were placed upright and incubated for 24 hrs at room temperature before freezing. After the 24 hrs incubation period, PAXgene blood tubes were stored at −80 °F until shipment. Frozen samples were sent to the PPMI Biorepository Core laboratories for RNA extraction. Blinded frozen samples were shipped in dry ice to Rosalind Franklin University of Medicine for the studies described herein.

### Quantitative Polymerase Chain Reaction Assays

Samples with RNA integrity values >7.0 and absorbance 260/280 between 1.2 and 3.0 were used in this study. One microgram of RNA was reverse transcribed into cDNA using a mix of random hexamer primers (High Capacity cDNA Synthesis Kit, Life Technologies, USA). RT-qPCR assays were performed using the DNA engine Opticon 2 Analyzer (Bio-Rad Life Sciences, Hercules, CA, USA). Each 25 microliters reaction contained Power SYBR Green (Life Technologies, USA) and primers at a concentration of 0.05 mM. Samples were run in triplicates and non-template control was used in all experiments. The geometric mean of two reference genes, glyceraldehyde-3-phosphate dehydrogenase (*GAPDH*) and phosphoglycerate kinase 1 (*PGK1*), were used to normalize for input RNA. Primer sequences for *PGK1* and *GAPDH* have been published elsewhere in refs 57 and 13. Primer sequences used for *NAMPT* are as follows: forward, 5′-CTATAAACAATATCCACCCAACACAAG-3′, reverse 5′-GTTTCCTCATATTTCACCTTCCTTAATT-3′. Samples were randomly distributed on PCR plates to avoid run-order bias. RT-qPCR amplification conditions have been reported in our previous studies^13^,^14^.

### Statistical Analysis

Statistical analysis was performed using STATISTICA 12 (StatSoft, OK, USA) and GraphPad Prism version 5 (GraphPad Software, Inc., CA, USA). A Student-t-test (unpaired, two tailed) was used to assess the differences between two groups and a chi-square test was used to analyze categorical data. Pearson correlation was performed for all correlations. The relative abundance of each biomarker was independently assessed using a general linear regression model adjusting for age, sex, and educational level. ROC analysis was performed to determine the diagnostic accuracy. We performed a forward step-wise linear discriminant analysis as demonstrated previously^13^,^14^ using our biomarker expression data, UPSIT scores and potential confounding variables including age and sex in STATISTICA 12 (StatSoft, OK, USA). A p-value of 0.05 or less was considered significant.

## Additional Information

**How to cite this article**: Santiago, J. A. *et al*. Integrative transcriptomic meta-analysis of Parkinson’s disease and depression identifies *NAMPT* as a potential blood biomarker for de novo Parkinson’s disease. *Sci. Rep.* **6**, 34579; doi: 10.1038/srep34579 (2016).

## Supplementary Material

Supplementary Information

## Figures and Tables

**Table 1:** Microarray datasets in blood of untreated PD and MDD patients.

GEO accession no.	No. of samples	Description	Platform	Reference
GSE54536	PD = 4; HC = 4	Untreated sporadic PD Patients (mean Hoehn and Yahr stage = 1)	Ilumina HT-12 V4	45
GSE72267	PD = 40; HC = 19	Untreated sporadic PD (mean Hoehn and Yahr stage = 1.4)	Affymetrix Human Genome U133A 2.0 Array	16
GSE32280	MDD = 8; HC = 8	Transcription profiling of blood from MDD and HC subjects.	Affymetrix Human Genome U133 Plus 2.0 Array	Unpublished
GSE46743	MDD = 69; HC = 91, males	Transcription profiling of blood from MDD and HC subjects.	Illumina HumanHT-12 V3.0	Unpublished

**Table 2:** Top 20 genes identified in meta-analysis of PD and MDD datasets.

Gene	Gene Description	Specificity	Overall Gene score
*NAMPT*	Nicotinamide phosphoribosyltransferase	4 out of 4	331.1
*LILRA5*	Leukocyte immunoglobulin-like receptor, subfamily A (with TM domain), member 5	3 out of 4	291.3
*LTBP3*	Latent transforming growth factor beta binding protein 3	3 out of 4	232.1
*EGR1*	Early growth response 1	2 out of 4	199.0
*CXCL5*	Chemokine (C-X-C motif) ligand 5	2 out of 4	197.5
*DUSP6*	Dual specificity phosphatase 6	2 out of 4	190.6
*IL1RN*	Interleukin 1 receptor antagonist	2 out of 4	185.2
*MNDA*	Myeloid cell nuclear differentiation antigen	2 out of 4	182.7
*GNB5*	Guanine nucleotide binding protein (G protein), beta 5	2 out of 4	182.6
*MS4A7*	Membrane-spanning 4-domains, subfamily A, member 7	2 out of 4	182.2
*MAN1C1*	Mannosidase, alpha, class 1C, member 1	2 out of 4	181.3
*CLEC7A*	C-type lectin domain family 7, member A	2 out of 4	179.0
*STAP1*	Signal transducing adaptor family member 1	2 out of 4	178.8
*CFLAR*	CASP 8 and FADD-like apoptosis regulator	2 out of 4	176.7
*LMO4*	LIM domain only 4	2 out of 4	176.4
*FOS*	FBJ murine osteosarcoma viral oncogene homolog	2 out of 4	176.0
*PATL2*	Protein associated with topoisomerase II homolog 2 (yeast)	2 out of 4	174.9
*RASSF4*	Ras association (RalGDS/AF-6) domain family member 4	2 out of 4	173.9
*IL18RAP*	Interleukin 18 receptor accessory protein	2 out of 4	173.1
*TPCN2*	Two pore segment channel 2	2 out of 4	172.9

Specificity indicates the number of datasets where the gene was significantly differentially expressed. The overall gene score is calculated from a non-parametric ranking in NextBio.

**Table 3:** Comparison of demographic and clinical characteristics between PD patients and HC.

Characteristic	HC (n = 101)	PD (n = 99)	P value^a^
Age, mean (SD) [95% CI], y	61 (10) [59–63]	63 (9) [61–65]	0.19
Female/male, No. (% male)	45/56 (55.4)	49/50 (50.5)	0.57^b^
Education, mean (SD) [95% CI], y	16.2 (2.9) [15.6–16.8]	15.1 (3.2) [14.4–15.7]	0.02
Disease duration, media (range), months	n/a	4 (1–36)	n/a
Hoehn and Yahr stage, mean (SD)	0.009 (0.09)	1.44 (0.50)	<0.001
MDS-UPDRS total	4.87 (4.41)	31.79 (12.41)	<0.001
MoCA, mean (SD) [95% CI]	28.23 (1.07) [28.02–28.44]	25.98 (2.53) [25.48–26.48]	<0.001
UPSIT score, mean (SD)[95% CI]	34.00 (4.86) [33.04–34.96]	21.08 (8.12) [19.46–22.70]	<0.0001
GDS score, mean (SD) [95% CI]	1.27 (1.89) [0.89–1.64]	2.15 (2.48) [1.64–2.67]	<0.007
SCOPA, mean (SD) [95% CI]	5.86 (3.28) [5.21–6.51]	9.27 (5.40) [8.16–10.38]	<0.0001

Abbreviations: CI = 95% confidence interval; GDS = Geriatric Depression Scale; HC = healthy controls; MoCA = Montreal Cognitive Assessment; MDS-UPDRS = Movement Disorder Society-sponsored revision of the Unified Parkinson’s Disease Rating Scale; PD = Parkinson’s disease; SCOPA = Scale for Outcomes in Parkinson’s disease for Autonomic Symptoms SD = standard deviation; y = years. UPSIT = University of Pennsylvania Smell Identification Test.

^a^Based on a Student t-test.

^b^Based on chi-square test (X^2^).
