# Supplementary material for: Integrative transcriptomic meta-analysis of Parkinson’s disease and depression identifies NAMPT as a potential blood biomarker for de novo Parkinson’s disease
Source: Sci Rep. 2016 Sep 29;6:34579. doi: 10.1038/srep34579 (PMC5041099; doi:10.1038/srep34579)
Supplement: Supplementary Information [file srep34579-s1.doc]

**Supplementary Information**

**Integrative transcriptomic meta-analysis of Parkinson’s disease and depression identifies *NAMPT* as a potential blood biomarker for de novo Parkinson’s disease**

Jose A. Santiago^1^, Alyssa M. Littlefield^1^ and Judith A. Potashkin^1*^

^1^The Cellular and Molecular Pharmacology Department, The Chicago Medical School, Rosalind Franklin University of Medicine and Science, North Chicago, IL, USA.

**Supplementary Table S1. Meta-analysis of blood microarrays from untreated PD and MDD patients using NextBio**. Specificity indicates the number of datasets where the gene was significantly differentially expressed. The overall gene score is calculated from a non-parametric ranking in NextBio.

| **Gene** | **Gene Description** | **Specificity** | **Overall Gene score** |
| --- | --- | --- | --- |
| *NAMPT* | Nicotinamide phosphoribosyltransferase | 4 out of 4 | 331.1 |
| *LILRA5* | Leukocyte immunoglobulin-like receptor, subfamily A (with TM domain), member 5 | 3 out of 4 | 291.3 |
| *LTBP3* | Latent transforming growth factor beta binding protein 3 | 3 out of 4 | 232.1 |
| *EGR1* | Early growth response 1 | 2 out of 4 | 199.0 |
| *CXCL5* | Chemokine (C-X-C motif) ligand 5 | 2 out of 4 | 197.5 |
| *DUSP6* | Dual specificity phosphatase 6 | 2 out of 4 | 190.6 |
| *IL1RN* | Interleukin 1 receptor antagonist | 2 out of 4 | 185.2 |
| *MNDA* | Myeloid cell nuclear differentiation antigen | 2 out of 4 | 182.7 |
| *GNB5* | Guanine nucleotide binding protein (G protein), beta 5 | 2 out of 4 | 182.6 |
| *MS4A7* | Membrane-spanning 4-domains, subfamily A, member 7 | 2 out of 4 | 182.2 |
| *MAN1C1* | Mannosidase, alpha, class 1C, member 1 | 2 out of 4 | 181.3 |
| *CLEC7A* | C-type lectin domain family 7, member A | 2 out of 4 | 179.0 |
| *STAP1* | Signal transducing adaptor family member 1 | 2 out of 4 | 178.8 |
| *CFLAR* | CASP8 and FADD-like apoptosis regulator | 2 out of 4 | 176.7 |
| *LMO4* | LIM domain only 4 | 2 out of 4 | 176.4 |
| *FOS* | FBJ murine osteosarcoma viral oncogene homolog | 2 out of 4 | 176.0 |
| *PATL2* | Protein associated with topoisomerase II homolog 2 (yeast) | 2 out of 4 | 174.9 |
| *RASSF4* | Ras association (RalGDS/AF-6) domain family member 4 | 2 out of 4 | 173.9 |
| *IL18RAP* | Interleukin 18 receptor accessory protein | 2 out of 4 | 173.1 |
| *TPCN2* | Two pore segment channel 2 | 2 out of 4 | 172.9 |
| *PASK* | PAS domain containing serine/threonine kinase | 2 out of 4 | 167.9 |
| *MYO6* | Myosin VI | 2 out of 4 | 166.8 |
| *FOSB* | FBJ murine osteosarcoma viral oncogene homolog B | 2 out of 4 | 165.6 |
| *DUSP1* | Dual specificity phosphatase 1 | 2 out of 4 | 164.9 |
| *APBA2* | Amyloid beta (A4) precursor protein-binding, family A, member 2 | 2 out of 4 | 163.3 |
| *CEACAM21* | Carcinoembryonic antigen-related cell adhesion molecule 21 | 2 out of 4 | 162.8 |
| *TNFRSF25* | Tumor necrosis factor receptor superfamily, member 25 | 2 out of 4 | 161.0 |
| *CETP* | Cholesteryl ester transfer protein, plasma | 2 out of 4 | 160.7 |
| *RLN1* | Relaxin 1 | 2 out of 4 | 160.1 |
| *SSH1* | Slingshot homolog 1 (Drosophila) | 2 out of 4 | 158.4 |
| *OASL* | 2'-5'-oligoadenylate synthetase-like | 2 out of 4 | 157.7 |
| *XAF1* | XIAP associated factor 1 | 2 out of 4 | 157.0 |
| *ADORA2B* | Adenosine A2b receptor | 2 out of 4 | 156.8 |
| *LPAR1* | Lysophosphatidic acid receptor 1 | 2 out of 4 | 156.8 |
| *TCF7L2* | Transcription factor 7-like 2 (T-cell specific, HMG-box) | 2 out of 4 | 155.4 |
| *KLF11* | Kruppel-like factor 11 | 2 out of 4 | 153.2 |
| *SLC11A2* | Solute carrier family 11 (proton-coupled divalent metal ion transporters), member 2 | 2 out of 4 | 152.4 |
| *FOXN2* | Forkhead box N2 | 2 out of 4 | 152.4 |
| *MGRN1* | Mahogunin ring finger 1, E3 ubiquitin protein ligase | 2 out of 4 | 151.3 |
| *CA6* | Carbonic anhydrase VI | 2 out of 4 | 151.1 |
| *QPCT* | Glutaminyl-peptide cyclotransferase | 2 out of 4 | 150.2 |
| *DPP4* | Dipeptidyl-peptidase 4 | 2 out of 4 | 150.1 |
| *SLC22A4* | Solute carrier family 22 (organic cation/ergothioneine transporter), member 4 | 2 out of 4 | 144.9 |
| *SNRNP70* | Small nuclear ribonucleoprotein 70kDa (U1) | 2 out of 4 | 142.5 |
| *LETM1* | Leucine zipper-EF-hand containing transmembrane protein 1 | 2 out of 4 | 138.2 |
| *SSRP1* | Structure specific recognition protein 1 | 2 out of 4 | 128.0 |
| *TMEM19* | Transmembrane protein 19 | 2 out of 4 | 128.0 |
| *RPA4* | Replication protein A4, 30kDa | 2 out of 4 | 127.1 |

**Supplementary Table S2. Diagnostic accuracy using *NAMPT* alone**

| **Diagnosis** | **Percent correct** | **PD** | **HC** |
| --- | --- | --- | --- |
| PD | 43.4 | 43 (True positives) | 56 (False positives) |
| HC | 72.3 | 28 (True negatives) | 73 (False negatives) |
| Total | 58 | 71 | 129 |

**Supplementary Table S3. Diagnostic accuracy using *COPZ1* alone**

| **Diagnosis** | **Percent correct** | **PD** | **HC** |
| --- | --- | --- | --- |
| PD | 49.5 | 49 (True positives) | 50 (False positives) |
| HC | 65.3 | 35 (True negatives) | 66 (False negatives) |
| Total | 58 | 84 | 116 |

**Supplementary Table S4. Diagnostic accuracy using *NAMPT* and *COPZ1***

| **Diagnosis** | **Percent correct** | **PD** | **HC** |
| --- | --- | --- | --- |
| PD | 43.3 | 43 (True positives) | 56 (False positives) |
| HC | 72.3 | 28 (True negatives) | 73 (False negatives) |
| Total | 58 | 71 | 129 |

**Supplementary Table S5. Summary of the step-wise linear discriminant analysis using *NAMPT* and *COPZ1.***

| Variable | Steps | Degrees of freedom | F to remove | P to remove | F to enter | P to enter | Effect status |
| --- | --- | --- | --- | --- | --- | --- | --- |
| sex | Step 1 | 1 |  |  | 0.48618 | 0.486456 | Out |
| age |  | 1 |  |  | 2.83255 | 0.093948 | Out |
| NAMPT |  | 1 |  |  | 11.65826 | 0.000775 | Entered |
| COPZ1 |  | 1 |  |  | 5.97125 | 0.015417 | Out |
| NAMPT | Step 2 | 1 | 11.65826 | 0.000775 |  |  | In |
| age |  | 1 |  |  | 3.01755 | 0.083931 | Out |
| sex |  | 1 |  |  | 0.60767 | 0.436603 | Out |
| COPZ1 |  | 1 |  |  | 1.32751 | 0.250646 | Out |

Supplementary Table S6. Diagnostic accuracy using RNA markers and UPSIT scores.

| **Diagnosis** | **Percent correct** | **PD** | **HC** |
| --- | --- | --- | --- |
| PD | 79.7 | 79 (True positives) | 20 (False positives) |
| HC | 92.1 | 93 (True negatives) | 8 (False negatives) |
| Total | 86 | 113 | 87 |

**Supplementary Table S7. Summary of the step-wise linear discriminant analysis using RNA markers and UPSIT scores.**

| Variable | Steps | Degrees of freedom | F to remove | P to remove | F to enter | P to enter | Effect status |
| --- | --- | --- | --- | --- | --- | --- | --- |
| UPSIT | Step 1 | 1 |  |  | 187.508 | 0 | Entered |
| sex |  | 1 |  |  | 0.4862 | 0.486456 | Out |
| age |  | 1 |  |  | 2.8326 | 0.093948 | Out |
| NAMPT |  | 1 |  |  | 11.6583 | 0.000775 | Out |
| COPZ1 |  | 1 |  |  | 5.9712 | 0.015417 | Out |
| UPSIT | Step 2 | 1 | 187.508 | 0 |  |  | In |
| sex |  | 1 |  |  | 2.0664 | 0.152165 | Out |
| age |  | 1 |  |  | 0 | 0.997396 | Out |
| NAMPT |  | 1 |  |  | 7.8025 | 0.005732 | Entered |
| COPZ1 |  | 1 |  |  | 3.0792 | 0.080855 | Out |
| UPSIT | Step 3 | 1 | 179.5795 | 0 |  |  | In |
| NAMPT |  | 1 | 7.8025 | 0.005732 |  |  | In |
| age |  | 1 |  |  | 0.0052 | 0.942734 | Out |
| sex |  | 1 |  |  | 2.2567 | 0.134643 | Out |
| COPZ1 |  | 1 |  |  | 0.4766 | 0.490785 | Out |

Supplementary Table S8. Diagnostic accuracy using UPSIT scores alone.

| **Diagnosis** | **Percent correct** | **PD** | **HC** |
| --- | --- | --- | --- |
| PD | 75.7 | 75 (True positives) | 24 (False positives) |
| HC | 93.1 | 7 (True negatives) | 94 (False negatives) |
| Total | 86 | 82 | 118 |
